# Downregulated MicroRNA-327 Attenuates Oxidative Stress–Mediated Myocardial Ischemia Reperfusion Injury Through Regulating the FGF10/Akt/Nrf2 Signaling Pathway

**DOI:** 10.3389/fphar.2021.669146

**Published:** 2021-05-07

**Authors:** Tao Zheng, Jun Yang, Jing Zhang, Chaojun Yang, Zhixing Fan, Qi Li, Yuhong Zhai, Haiyin Liu, Jian Yang

**Affiliations:** ^1^ Department of Cardiology, the First College of Clinical Medical Science, China Three Gorges University, Yichang, China; ^2^ HuBei Clinical Research Center for Ischemic Cardiovascular Disease, Yichang, China; ^3^ Yichang Key Laboratory of Ischemic Cardiovascular and Cerebrovascular Disease Translational Medicine, Yichang, China; ^4^ Institute of Cardiovascular Disease, China Three Gorges University, Yichang, China; ^5^ Department of Cardiology, the People’s Hospital of Three Gorges University, Yichang, China

**Keywords:** myocardial ischemia/reperfusion injury, oxidative stress, microRNA-327, FGF10, apoptosis

## Abstract

Although miR-327 had a protective effect on cardiomyocytes as described previously, the potential mechanism still needs further exploration. The aim of this study was to investigate the role and mechanism of miR-327 on oxidative stress in myocardial ischemia/reperfusion injury (MI/RI) process. Oxidative stress and cardiomyocytes injury were detected in rat model of MI/RI, hypoxia/reoxygenation (H/R), and tert-butyl hydroperoxide (TBHP) model of H9c2 cells. *In vitro*, downregulation of miR-327 inhibited both H/R- and TBHP-induced oxidative stress, and suppressed apoptosis. Meanwhile, fibroblast growth factor 10(FGF10) was enhanced by miR-327 knocked down, followed by the activation of p-PI3K and *p*-Akt, and the translocation of Nrf2. However, miR-327 overexpression performed with opposite effects. Consistent with the results *in vitro*, downregulation of miR-327 attenuated reactive oxygen species (ROS) generation as well as intrinsic apoptosis, and alleviated I/R injury. In conclusion, inhibition of miR-327 improved antioxidative ability and myocardial cell survival *via* regulating the FGF10/Akt/Nrf2 pathway.

## Introduction

Rapid reperfusion therapy can effectively restore the oxygen and nutrient supply in the ischemic area and avoid further degeneration of the infarcted myocardium in ischemic heart disease (IHD) patients (Anderson and Morrow, 2017). Meanwhile, inflammation, excessive ROS, apoptosis, and other pathological can also happen in the reperfusion area, which indicates that myocardial ischemia reperfusion injury (MIRI) occurred (Sanchez-Hernandez et al., 2020). The ROS outbreak at the early period of reperfusion is the driving factor leading to I/R injury (Cadenas, 2018). Its burst triggers mitochondrial rupture-activated apoptosis and increased inflammatory cytokine signaling, as well as may alter matrix metalloproteinases (MMPs) activity (Cadenas, 2018; Bugger and Pfeil, 2020). Therefore, minimizing or even reversing MIRI based on the benefits of reperfusion therapy, especially at oxidative stress, may have great clinical prospect.

MicroRNAs (miRNAs) have been shown to cross talk with oxidative stress in a variety of diseases (Hathaway et al., 2018; Konovalova et al., 2019). Some miRNAs have been reported to mediate oxidative stress through directly targeting antioxidant genes (e.g., Trx1 and Sirt) or indirectly interfering with pro-survival signaling pathways (e.g., Akt and IGF-1) and redox transcription factors (e.g., p53, NF-κB, and Nfr2) (Lin, 2019; Climent et al., 2020). Conversely, oxidative stress also affects miRNA expression (Xiao et al., 2015). Our previous work has reported that miR-327 involved in intrinsic apoptosis during MI/RI (Li et al., 2020). It is well known that the excessive ROS is a key factor in the initiation of intrinsic apoptosis (Hossain et al., 2020). However, little is known about miR-327 and oxidative stress in MIRI. Interestingly, Fischer et al. demonstrated that FGF10, as a target gene of miR-327, specifically inhibits the FGF10-triggered fibroblast growth factor receptor (FGFR)–Akt signaling axis in the white fat browning study (Fischer et al., 2017). Besides, in a study of peripheral nerve regeneration, FGF10 treatment inhibits peripheral nerve injury–induced oxidative stress by activating PI3k/Akt signaling in the sciatic nerve (Dong et al., 2019). Whether miR-327 can regulate the FGF10/Akt signaling pathway participated in oxidative stress of MIRI is far from clear yet.

Accordingly, this study intended to explore the effects and underlying mechanisms of miR-327 on oxidative stress in myocardial I/R based on the H/R and TBHP models in H9c2 cells, as well as the MIRI model in rats, respectively.

## Materials and Methods

### Cell Culture and Treatment

The rat H9c2 cardiomyocyte cell line was procured from the Cell Bank of China Science Academy (Shanghai, China). All cells were cultured in a high-glucose Dulbeccoʼs modified Eagleʼs medium (HyClone, Thermo Fisher Scientific, Inc., Wilmington, DE) containing 100 μg/ml streptomycin, 100 U/ml penicillin, and 10% fetal bovine serum (HyClone; Thermo Fisher Scientific, Inc.) at 37°C in a 5% CO_2_ incubator.

As described previously, the AnaeroPack method was used to mimic the MI/RI model *in vitro* (Masaki et al., 2005; Wei et al., 2015). The H9c2 cells cultured in a plate filled with low-glucose DMEM were placed in a sealed container with an AnaeroPack (Mitsubishi Gas Company, Tokyo, Japan). After incubating for 4 h under hypoxic conditions at 37°C, the cells were reoxygenated for 2 h under normoxic conditions (Bian et al., 2018).

For experiments, TBHP, which has a higher stability than hydrogen peroxide (H_2_O_2_), was used to induce oxidative damage (Slamenova et al., 2013). Bi et al. (Bi et al., 2018) treated H9c2 cells with 75 μM TBHP for 4 h, resulting in a decrease in cell viability and accompanied by the expression changes of Bax and Bcl-2 proteins which may be associated with the ROS outbreak. Based on it, TBHP was diluted with DMEM to 50 μM and stimulated the H9c2 cells for 4 h.

### Cell Transfection

The adenoviral vectors encoding miR-327 harboring RNAi sequence (Ad-miR-327i group), miR-327 (Ad-miR-327 group), and control-enhanced green fluorescent protein (Ad-NC group) were constructed and provided by GeneChem (Shanghai, China) (Yang et al., 2018). The H9c2 cells were transfected with adenoviral vectors for 48 h before H/R or TBHP treatment, respectively (Li et al., 2020).

### Cell Viability Assay

The Cell Counting Kit-8 (CCK-8) assay (Dojindo, Tokyo, Japan) was utilized to examine cell viability in each treatment. The H9c2 cells (5×10^3^ cells/well) were plated in 96-well culture plates, exposed to H/R or TBHP with or without adenoviral transfection.

### Animals

The experiments were approved by the Ethics Committee for Animal Experimental Center of China Three Gorges University and were performed in adherence with the U.S. National Institutes of Health guidelines. Healthy adult male-specific pathogen-free SD rats (weighing 230 ± 10 g) were purchased from the Animal Experimental Center of China Three Gorges University (Yichang, China).

### Myocardial Ischemia/Reperfusion Protocol

Rats were randomized into five groups: 1) sham group: normal nonischemic, 2) I/R group: myocardial I/R, 3) Ad-NC group: myocardial I/R with Ad-EGFP-NC, 4) Ad-miR-327i group: myocardial I/R with Ad-miR-327-inhibition, and 5) Ad-miR-327 group: myocardial I/R with Ad-miR-327.

Prior to myocardial ischemia/reperfusion operation, the cardiac rats were transfected with recombinant adenovirus by cardiac apex injection. In short, rats were anesthetized with 3% sodium pentobarbital (40 mg/kg, IP) and connected to a small animal ventilator. The heart was exposed through a left thoracic incision, and the pericardium was torn out gently. The solution of recombinant adenoviruses vectors (1.0 × 10^10^ PFU/ml) or an equal volume of normal saline (NS) was injected with five needles in different regions of the apical apex, respectively. After that, the chest was closed rapidly, the skin was sutured, and rats were returned to their cage when they woke up.

After three days of careful feeding with intramyocardial injection, the MI/RI model was performed (Wang et al., 2016). The rat’s chest was opened in the same way as mentioned above. Passing a 6–0 silk suture under the origin of the left anterior descending coronary artery (LAD), and then placing a medical latex tube between the ligature and LAD, a slipknot was made to induce myocardial ischemia for 30 min. Rats in the sham group were also passed a suture under the LAD but without occlusion. After 2 h of reperfusion, heart specimens and blood sample were collected under anesthesia (Li et al., 2018).

### Determination of Myocardial Infarct Size

After 2 h reperfusion, the hearts were taken out immediately under anesthesia and flushed with 0.9% saline, and then the hearts were placed in a refrigerator at −20°C for 20 min. The slices were placed in 1.5% 2, 3, 5-triphenyltetrazolium chloride (TTC, Sigma-Aldrich, United States) solution at 37°C in the dark for 15 min and fixed in tissue fixative overnight. Image-Pro Plus 7.0 software was used to calculate the infarct area, and the ratio of infarct area (IA)/left ventricle (LV) area was obtained. Cardiac tissue was collected from the reperfused rats and fixed with 4% paraformaldehyde for 24 h. Routine paraffin sections were made and Hematoxylin Eosin (HE) staining was performed to observe the histopathological changes of myocardium in the reperfusion area of rats in each group.

### Biochemical Index Analysis

The activities of the antioxidant enzymes SOD and GSH-Px, and the levels of MDA in the heart tissues or H9c2 cell homogenates were determined following the manufacturer’s protocol (Beyotime Biotechnology, Shanghai, China). The activities of lactate dehydrogenase (LDH) both in serum and culture supernate were assessed using the LDH kits (Nanjing Jiancheng Bioengineering Institute, Nanjing, China) according to the instructions.

### ROS Detection

Intracellular ROS generation was quantified with a Fluorometric Intracellular ROS Kit (MAK144, Sigma-Aldrich), which was used to assess mitochondrial damage and to measure intracellular ROS production (Al-Azab et al., 2020). The H9c2 cells were treated with H/R or TBHP, the culture media were disposed, and then, 100 uL/well of master reaction mix were added into the cell plate and incubated at 37°C for 30 min. Three visual fields were randomly taken from each well, then images were taken by a Nikon Confocal Laser Microscope A1R+ (Tokyo, Japan), and the fluorescence mean density was measured by Image-Pro Plus 7.0 software. For flow cytometry, the H9c2 cells were harvested, centrifuged, and washed once with phosphate-buffered saline (PBS) after H/R treatment. 2 uL of ROS Detection Reagent Stock Solution was added to the cell medium and incubated for 30 min, then the supernatant was removed, and cells were suspended in sheath fluid. The flow cytometer (BD verse, US) was used to quantify the DCF fluorescence intensity of intracellular ROS.

Fluorescent probe dihydroethidium (DHE, Invitrogen, Carlsbad, CA, United States) was used to evaluate superoxide production in the reperfusion area of the left ventricle myocardium. About 10 μm sections were cut from frozen myocardial tissue and then incubated with 10 μM DHE at 37°C for 30 min in a dark environment. For each group, three myocardial sections were obtained, and images were obtained with a fluorescence microscope, and then, three visual fields were randomly selected in each section for quantitative analysis using Image-Pro Plus 7.0 (Zeng et al., 2020).

### Apoptosis Detection

The Annexin V-APC/propidium iodide dual staining was used to detect apoptosis of H9c2 cells after TBHP treat according to the manufacturer’s recommendations. The FCM on BD FACSCalibur (Becton Dickinson Co., San Jose, CA) was used to quantify the apoptosis ratio.

### Quantitative Real-Time Polymerase Chain Reaction

The total RNA of cells and tissues was extracted with a TRIzol Kit (Invitrogen), and the first strand cDNA was reversely transcribed according to the instructions of a cDNA Reverse Transcription Kit (Takara, China). The primers were synthesized and provided by RiboBio (Guangzhou, Chian). SYBR PCR Master Mix (Roche) was used for qRT-PCR. The PCR program setup referred to our previous study (Li et al., 2020). The internal reference of miR-327 is U6 and that of FGF10 is GAPDH. The primer sequences were as follows:

Rno-miR-327 loop-primer 5′-GTCGTATCCAGTGCAG GGTCCGAGGTATTCGCACTGGATACGACACCCTCAT-3′
Forward primer 5′-TGC​GCC​CTT​GAG​GGG​CAT​G-3′, Reverse primer 5′-CCA​GTG​CAG​GGT​CCG​AGG​TAT​T-3’;
U6: Forward primer 5′-CGC​TTC​GGC​AGC​ACA​TAT​AC-3′, Reverse primer 5′-AAA​TAT​GGA​ACG​CTT​CAC​GA-3’;
FGF10: Forward primer 5′-CAA​CGG​CAG​GCA​AAT​GTA​TG-3′, Reverse primer 5′-AGG​AAG​TGA​GCG​GAG​GTG​TT-3’;
GAPDH: Forward primer 5′-GAA​CGG​GAA​GCT​CAC​TGG-3′,
Reverse primer 5′-GCC​TGC​TTC​ACC​ACC​TTC​T-3’.

### Western Blotting

The total protein and nucleus protein of cells and cardiac tissue were extracted according to the manufacturer’s protocol (Beyotime Biotechnology, China). After the protein concentration was determined, it was loaded and separated by sodium dodecyl sulfate polyacrylamide gel electrophoresis, transferred to polyvinylidene fluoride membrane, and then, it was incubated with antibodies against FGF10 (1:2000; Abcam, Cambridge, United Kingdom), PI3K(1:5000; Proteintech Group, Inc, Wuhan, China), phospho-PI3K(1:1000; Cell Signaling Technology, Germany), total-Akt (1:1000; Proteintech Group), *p*-Akt (1:2000; Proteintech Group), Nrf2(1:500; Proteintech Group), Histone-H3 (1:1000; Proteintech Group), HO-1 (1:1000; Proteintech Group), Bax (1:1000; Cell Signaling Technology, Germany), Bcl-2 (1:1000; Abcam), cytochrome-c (Cyt-c) (1:1000; Affinity, Cincinnati), cleaved caspase-9 (1:500; Proteintech Group), cleaved caspase-3 (1:1000; Abcam), and GAPDH (1:10,000; Proteintech Group) overnight at 4°C. The membrane was rinsed with Tris-buffered saline with 0.1% Tween 20 and then incubated with horseradish peroxidase–conjugated secondary antibodies for 2 h. Subsequently, the ECL detection system was used for analysis.

### Statistical Analysis

SPSS 19.0 software (IBM Corp, Armonk, NY) was used for the statistical significance between groups, and all data were presented as the mean ± SD. The normality of the data was tested by the Shapiro–Wilk method. An unpaired Student’s *t* test was used for comparison between two groups. Comparisons of more than two groups were performed by one-way analysis of variance, followed by the Tukey’s multiple comparison test. The value of *p* < 0.05 was considered statistically significant.

## Results

### H/R Induced Oxidative Stress and miR-327 Expression

**FIGURE 1:**
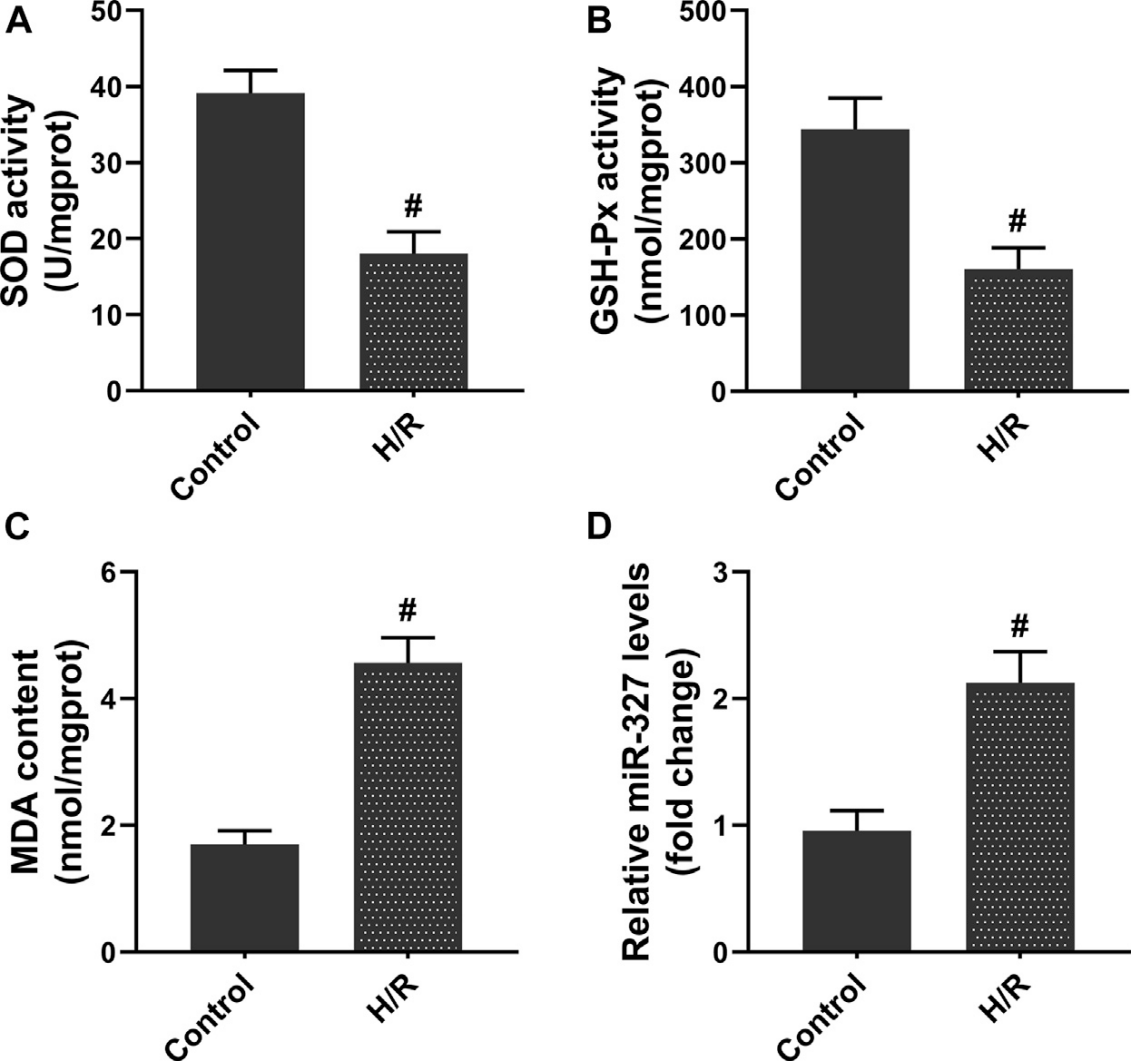
H/R induced oxidative stress, and the expression of miR-327 was upregulated in H9c2 cells. The cellular redox state in H/R H9c2 cells was assessed by SOD **(A)**, GSH-Px **(B)** activities, and MDA **(C)** content. **(D)** Relative expression of miR-327 in H/R H9c2 cells. Data are expressed as the mean ± SD; *n* = 3; #*p* < 0.05 compared to the Control group.

First, to assess the oxidative stress after H/R in H9c2 cells, the biochemical indices including SOD, GSH-Px, and MDA were detected. After 4 h of hypoxia and reoxygenation for 2 h (Bian et al., 2018), the intracellular SOD and GSH-Px activities were remarkably decreased, while MDA production dramatically increased compared with the Control group (Figures 1A–C). Moreover, the qPCR assay showed that exposure to H/R obviously increased the miR-327 expression (Figure 1D). The results obtained implied that miR-327 might involve in regulating oxidative stress under H/R condition.

### MiR-327 Participated in Oxidative Stress Induced by H/R *in Vitro*

**FIGURE 2:**
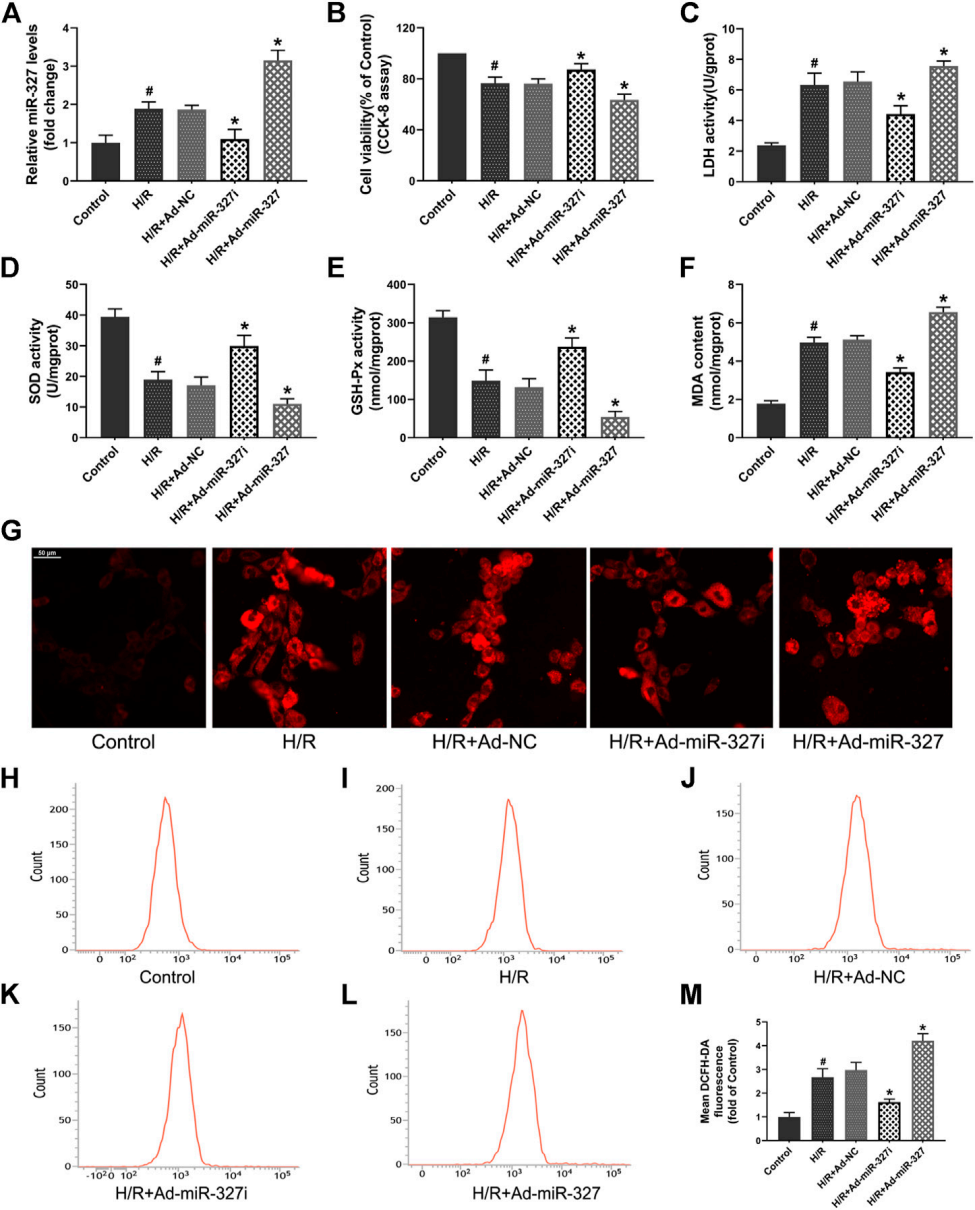
Effects of miR-327 on oxidative stress in H/R H9c2 cells **(A)**. qRT-PCR detected the miR-327 expression levels in each group. The cell damage was determined by CCK-8 assay **(B)** and LDH activity **(C)**. Oxidative stress indexes of SOD **(D)**, GSH-Px **(E)**, and MDA **(F)** were detected after H/R treatment. The confocal laser microscopy shows images of intracellular ROS staining **(G)**. The flow cytometry analysis was used for quantitative analysis of DCFH-DA fluorescence of intracellular ROS in each group **(H-L)**, and the results were expressed in bar graphs **(M)**. All values are presented as the mean ± SD (*n* = 3); #*p* < 0.05 versus the Control group; *p < 0.05 compared to the Ad-NC group.

To gain some insight into the role of miR-327 in oxidative stress induced by H/R, we transfected an adenovirus vector containing miR-327-RNAi and pre-miR-327 in H9c2 cells before treatment. The qPCR result showed that the miR-327 expression could be successfully decreased by Ad-miR-327i or over-expressed by Ad-miR-327 transfection in H9c2 cells under H/R (Figure 2A).

The cell viability, evaluated by the CCK-8 assay, was obviously decreased in H/R-treated H9c2 cells, while Ad-miR-327i reduced this effect. The LDH activity in the Ad-miR-327i group was significantly decreased. The Ad-miR-327 group showed severe cell damage, indicated by the CCK-8 and LDH assay, compared with the Ad-NC group (Figures 2B,C). Meanwhile, the SOD and GSH-Px activities were markedly increased in the Ad-miR-327i group, and MDA levels were significantly decreased compared with the Ad-NC group. In the Ad-miR-327 group, however, they showed inverse changes (Figures 2D–F). Excessive ROS, generated during H/R treatment, was considered to be the determining factor for oxidative stress, so the ROS levels were measured by a confocal laser microscope and flow cytometry. The intracellular ROS staining revealed that Ad-miR-327i suppressed the ROS production induced by H/R in H9c2 cells, while Ad-miR-327 abolished the protective effect (Figure 2G-M). Accordingly, these data suggest that miR-327 downregulation plays a cardioprotective role by mediating oxidative stress under H/R condition.

### miR-327 Regulated FGF10 and Altered the PI3K/Akt/Nrf2 Signaling Pathway Under H/R Stimulation

**FIGURE 3:**
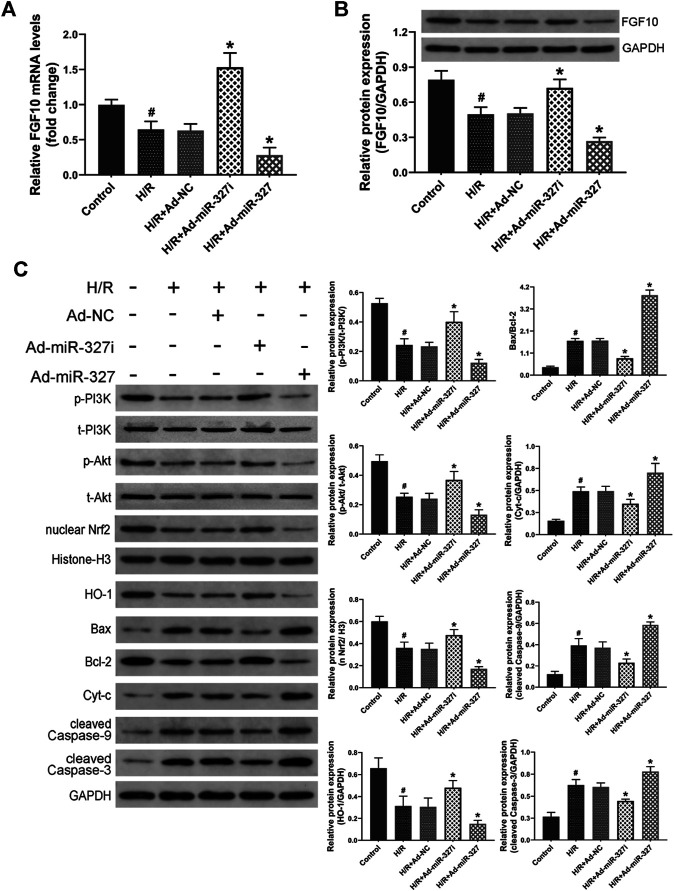
Effects of miR-327 on the FGF10/Akt/Nrf2 signaling pathway in H/R H9c2 cells. FGF10 mRNA and protein levels were measured by the qRT-PCR and the Western blot **(A-B)**. FGF10 downstream protein p-PI3K and t-PI3K, *p*-Akt and t-Akt, antioxidant-related protein nucleus Nrf2 and HO-1, apoptosis-related protein Bax, Bcl-2, Cyt-c, cleaved caspase-9, and cleaved caspase-3 were detected by Western blot analysis in different groups of H9c2 cells **(C)**. Data are expressed as the mean ± SD (*n* = 3); #*p* < 0.05 versus the Control group; *p < 0.05 versus the Ad-NC group.

A previous study has demonstrated that FGF10 was a target of miR-327, and miR-327 can regulate the FGF10/Akt signal pathway in beige adipocyte (Fischer et al., 2017). Whether miR-327 could alter the FGF10 expression in cardiomyocytes under H/R condition is still unknown. Therefore, we initially examined the expressions of FGF10 though qPCR and Western blots. H/R stimulation induced obviously decreased levels of FGF10 expression, Ad-miR-327i enhanced both mRNA and protein expressions of FGF10, but Ad-miR-327 potently inhibited the FGF10 expression compared with the Ad-NC group (Figures 3A,B). Then, we investigated whether the PI3K/Akt/Nrf2 pathway was involved in miR-327–mediated oxidative stress induced by H/R. The Western blot analysis showed that Ad-miR-327i increased the phosphorylation of PI3K and Akt in H/R-treated H9c2 cells, whereas Ad-miR-327 further blocked the activation of PI3K/Akt signaling. Meanwhile, Ad-miR-327i also increased nuclear Nrf2 and HO-1 under H/R, while Ad-miR-327 reversed these changes compared with the Ad-NC group (Figure 3C).

Apoptosis, especially intrinsic apoptosis, was strongly associated with oxidative stress (Cadenas, 2018), so we mainly detected the intrinsic apoptosis-related proteins. H/R induced an increase of Bax/Bcl-2, Cyt-c, cleaved caspase-9, and cleaved caspase-3. But Ad-miR-327i significantly reduced the levels of these proteins compared with the Ad-NC group (Figure 3C).

### The miR-327 Expression Was Elevated Under TBHP Stimulation

**FIGURE 4:**
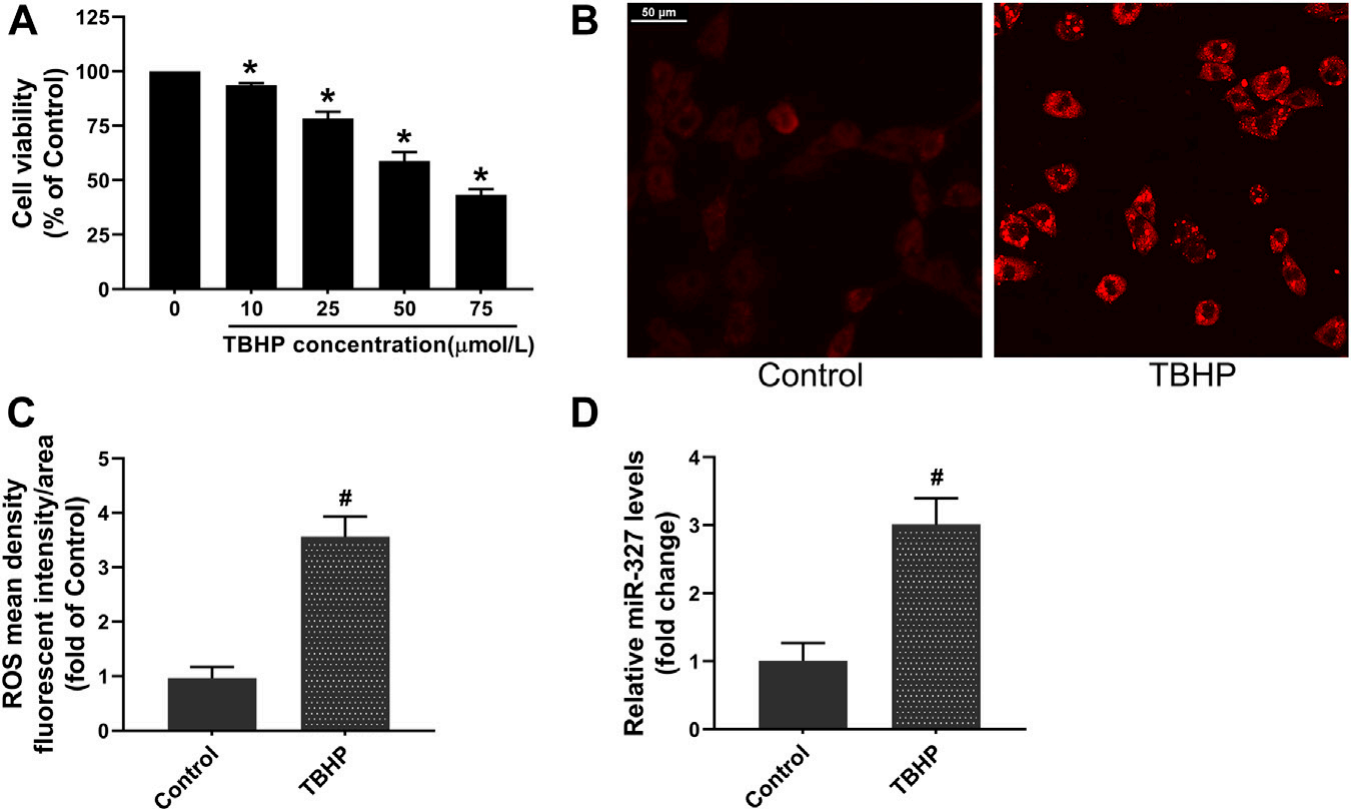
TBHP induced oxidative damage and promoted miR-327 expression in H9c2 cells. **(A)** Cell viability with different concentrations of TBHP. The effect of 50 μM TBHP on intracellular ROS production in H9C2 cells, images were obtained by confocal laser microscopy **(B)** and analyzed by Image-Pro Plus **(C)**. **(D)** Relative expression of miR-327 in cells tested by qRT-PCR. Data are expressed as the mean ± SD; *n* = 3; *p < 0.05 versus the normal group; #*p* < 0.05 compared to the Control group.

TBHP is often used to establish oxidative damage models in cells, and different TBHP concentrations can trigger different cell death modalities, such as apoptosis and necrosis (Wang et al., 2015). Bi et al. (Bi et al., 2018) incubated H9c2 cells with a relatively low TBHP concentration, resulting in a decrease of cell viability to 54.61 ± 4.78%, and induced apoptosis. Based on this, we carried out a dose-dependent manner experiment to explore the effect of TBHP concentration on cell viability. The CCK-8 assay demonstrated that 50 μM TBHP decreased cell viability to 58.84 ± 4.10%. Moreover, the ROS production in H9c2 cells was increased about 3.5-fold after 50 μM TBHP treatment (Figures 4B,C),which was considered to be an appropriate concentration of TBHP for establishing oxidative stress and apoptosis in H9c2 cells in subsequent experiments. Interestingly, the qPCR assay showed that the expression of miR-327 was significantly upregulated nearly 3-fold in response to TBHP (Figure 4D).

### Downregulation of miR-327 Attenuated TBHP-Induced Oxidative Stress

**FIGURE 5:**
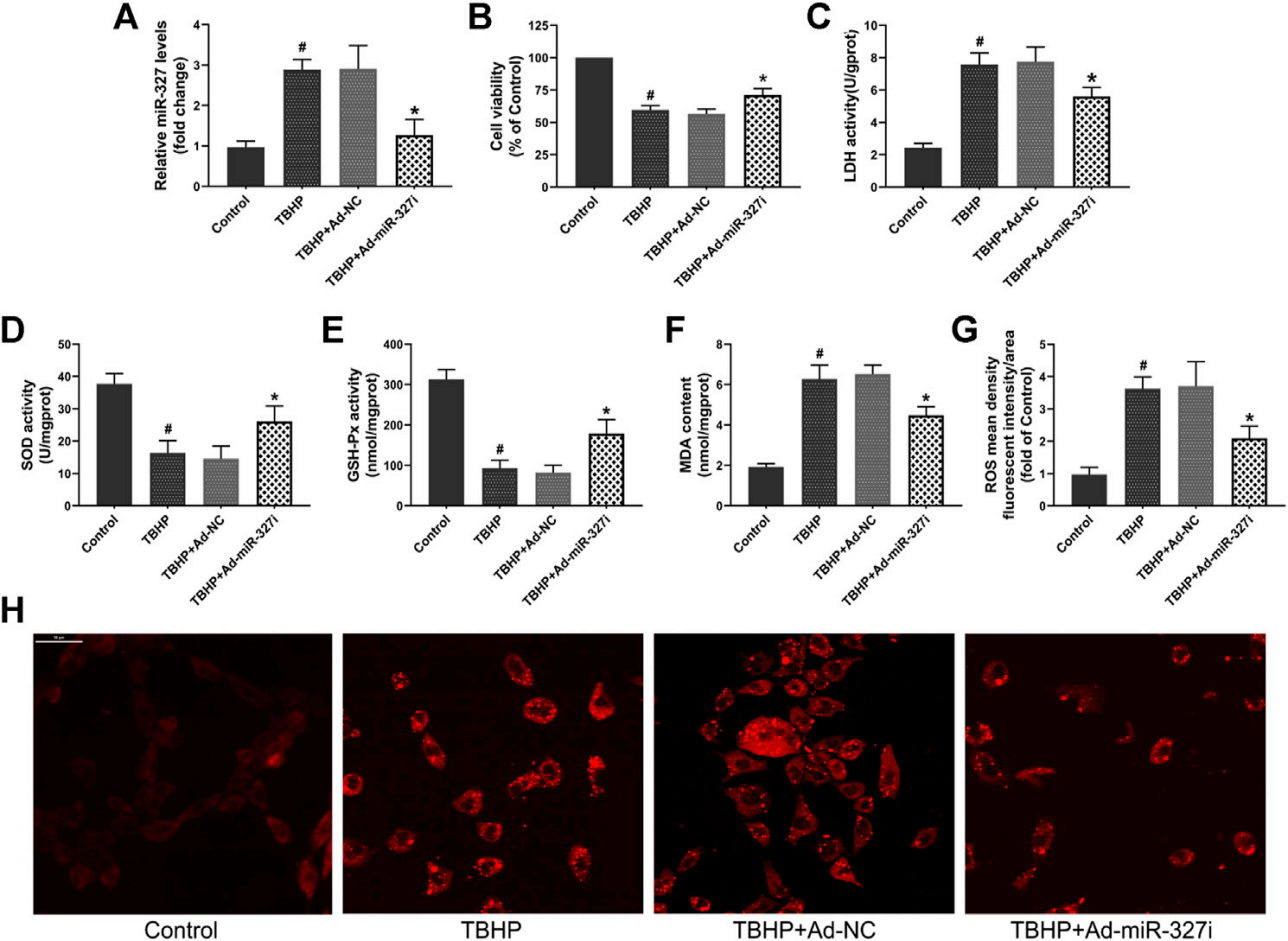
Effects of downregulation of miR-327 on TBHP induced oxidative damage in H9c2 cells. **(A)** Relative expression of miR-327 in H9c2 cells after 4 h of TBHP treatment. Cell viability **(B)** and LDH activity **(C)** were used to evaluate cell damage. The levels of SOD **(D)**, GSH-Px **(E)**, and MDA **(F)** in TBHP-treated H9c2 cells. Representative images of intracellular ROS staining **(H)** and ROS mean density fluorescent intensity was analyzed by Image-Pro Plus **(G)**. Date are expressed as the mean ± SD (*n* = 4); #*p* < 0.05 versus the Control group; *p < 0.05 versus the Ad-NC group.

To evaluate whether downregulation of miR-327 can alleviate TBHP-induced oxidative stress, we transfected an Ad-miR-327i adenovirus vector in H9c2 cells. The qPCR assay confirmed that in the Ad-miR-327i group, the expression of miR-327 was significantly reduced compared to that in the Ad-NC group (Figure 5A). The cell viabiltiy was restored to 71.34 ± 4.82% in the Ad-miR-327i group, and the LDH level was lower than that in the Ad-NC group (Figures 5B,C). Meanwhile, Ad-miR-327i increased the activities of SOD (Figure 5D) and GSH-Px (Figure 5E), and decreased the MDA (Figure 5F) level under TBHP condition. Moreover, TBHP-induced ROS production was suppressed by Ad-miR-327i compared to Ad-NC (Figures 5G,H). These findings indicated that downregulation of miR-327 exerted protective cardiomyocytes from oxidative stress under TBHP stimulation.

### Downregulation of miR-327 Suppressed TBHP-Induced Apoptosis

**FIGURE 6:**
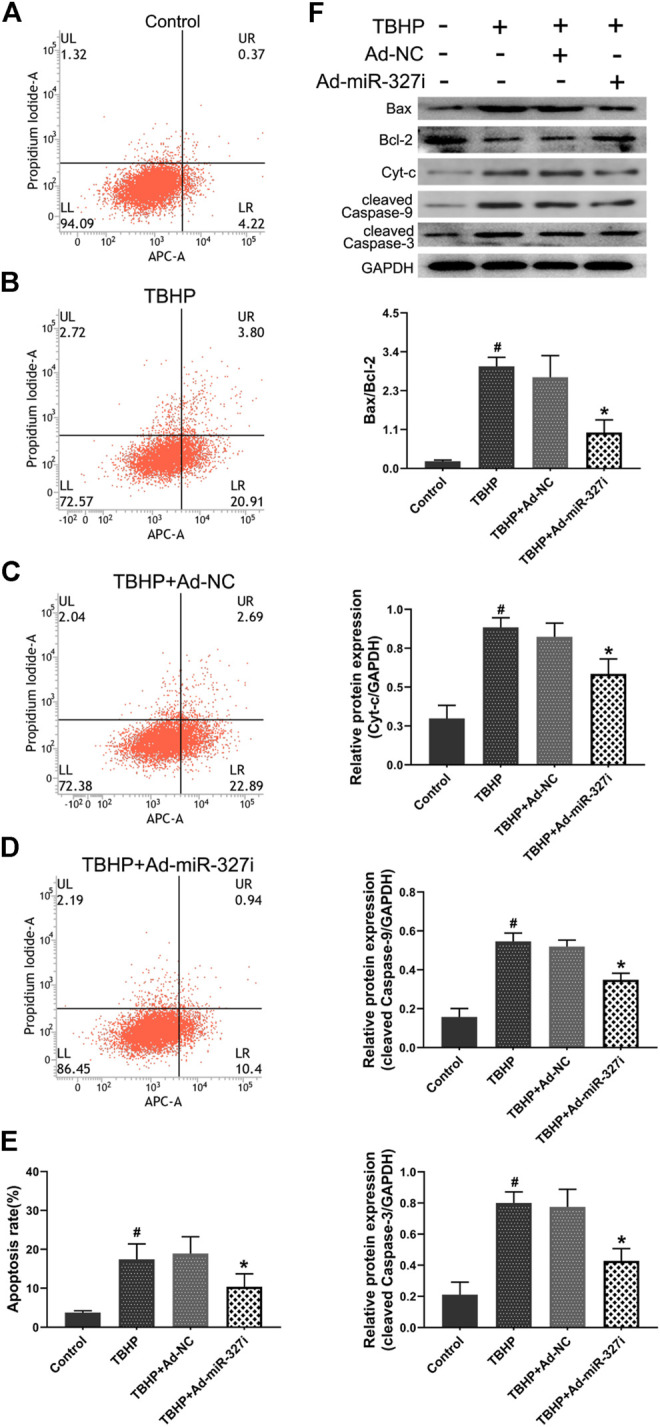
Effects of downregulation of miR-327 on apoptosis in TBHP treated H9c2 cells. The AV-APC/PI double-staining assay was performed to detect apoptosis in H9c2 cells after TBHP treatment **(A-D)**, and the relative apoptotic indexes in each group were expressed in bar graphs **(E)**. Apoptosis-related protein Bax, Bcl-2, Cyt-c, cleaved caspase-9, and cleaved caspase-3 expression levels were detected by the Western blot analysis in H9c2 cells **(F)**. Data are expressed as the mean ± SD (*n* = 3); #*p* < 0.05 versus the Control group; *p < 0.05 versus the Ad-NC group.

Then, we explored the effect of downregulation of miR-327 on TBHP-induced apoptosis in H9c2 cells. The apoptosis rate was increased from 3.75 ± 0.47% to 17.41 ± 4.01% after TBHP treatment (Figures 6A,B,E). Ad-miR-327i inhibited this malignant increase (18.90 ± 4.34% vs. 10.37 ± 3.35%) compared to Ad-NC (Figures 6C–E). We also examined the expression of intrinsic apoptosis–related proteins Bax, Cyt-c, cleaved caspase-9, cleaved caspase-3, and antiapoptotic molecule Bcl-2. TBHP treatment typically enhanced the Bax/Bcl-2, Cyt-c, cleaved caspase-9, and the cleaved caspase-3 protein expression. Ad-miR-327i dramatically attenuated the expression levels of these proteins compared to Ad-NC under TBHP (Figure 6F).

### Downregulation of miR-327 Enhanced FGF10/Akt/Nrf2 Signaling Transduction in Response to TBHP

**FIGURE 7:**
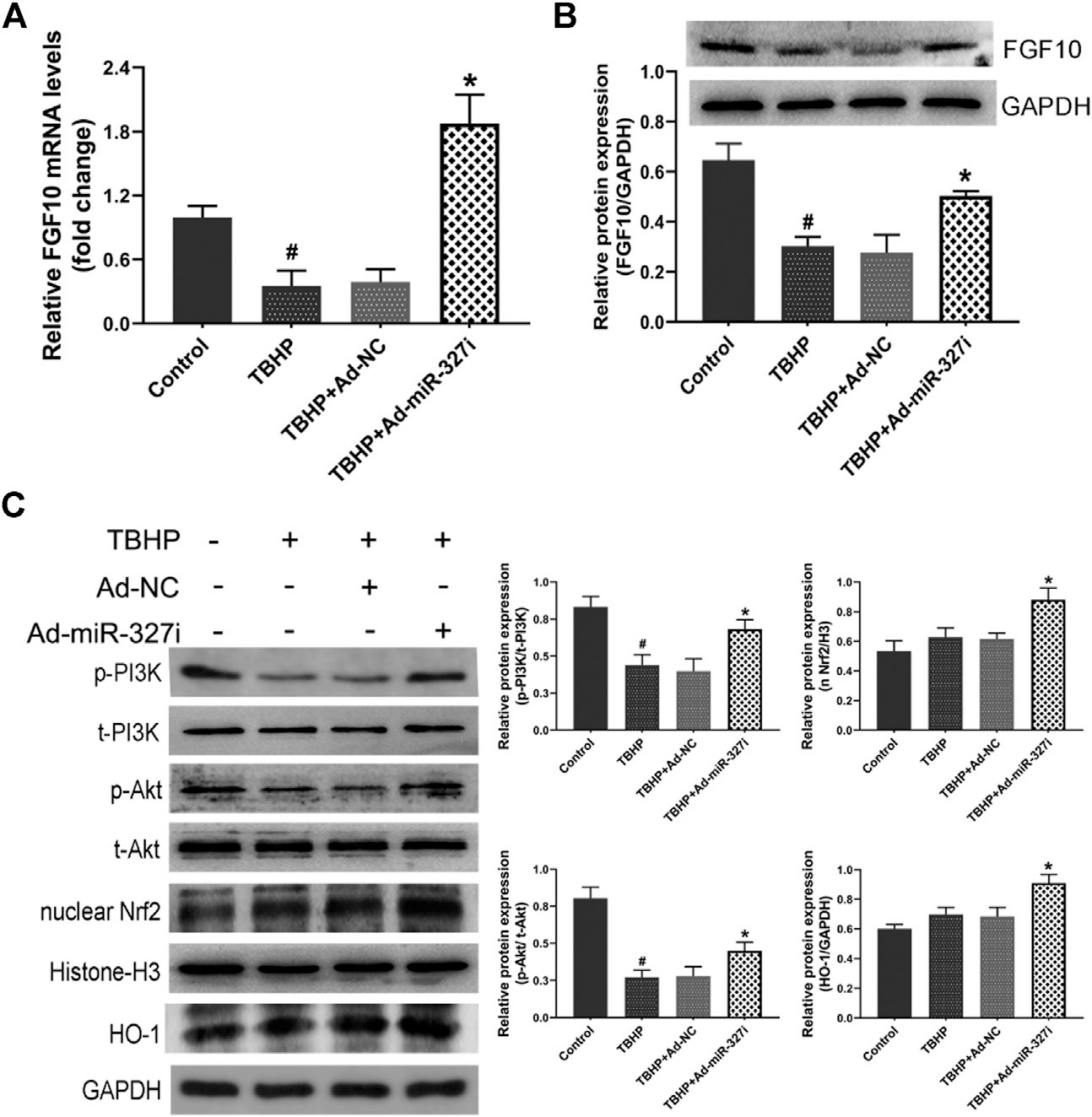
Effects of downregulation of miR-327 on the FGF10/Akt/Nrf2 signaling pathway in TBHP-treated H9c2 cells. FGF10 mRNA and protein levels were detected by qRT-PCR and Western blot analysis after TBHP treatment **(A, B)**. Downstream protein p-PI3K and t-PI3K, t-Akt and *p*-Akt, antioxidant-related protein nucleus Nrf2, and HO-1 were measured by the Western blot analysis in H9c2 cells **(C)**. Data are expressed as the mean ± SD (*n* = 3); #*p* < 0.05 versus the Control group; *p < 0.05 versus the Ad-NC group.

To investigate the molecular mechanism of downregulation of miR-327 on the enhanced antioxidant effect, the FGF10/Akt/Nrf2 signaling pathway was examined by the Western blot. The mRNA and protein levels of FGF10 were decreased under TBHP stimulation, which were obviously increased by miR-327 knockdown (Figures 7A,B). The phosphorylation of PI3K and Akt was reduced under TBHP condition, while the expressions of nuclear Nrf2 and HO-1 were not significantly altered. Moreover, Ad-miR-327i not only elevated p-PI3K/t-PI3K and *p*-Akt/t-Akt but also further promoted nuclear Nrf2 and HO-1 expressions compared to Ad-NC in response to TBHP (Figure 7C). Taken together, these data suggested that the enhanced antioxidant effect of downregulation of miR-327 may be associated with the activation of the FGF10/Akt/Nrf2 pathway.

### MiR-327 Participated in Oxidative Stress Induced by MI/RI

**FIGURE 8:**
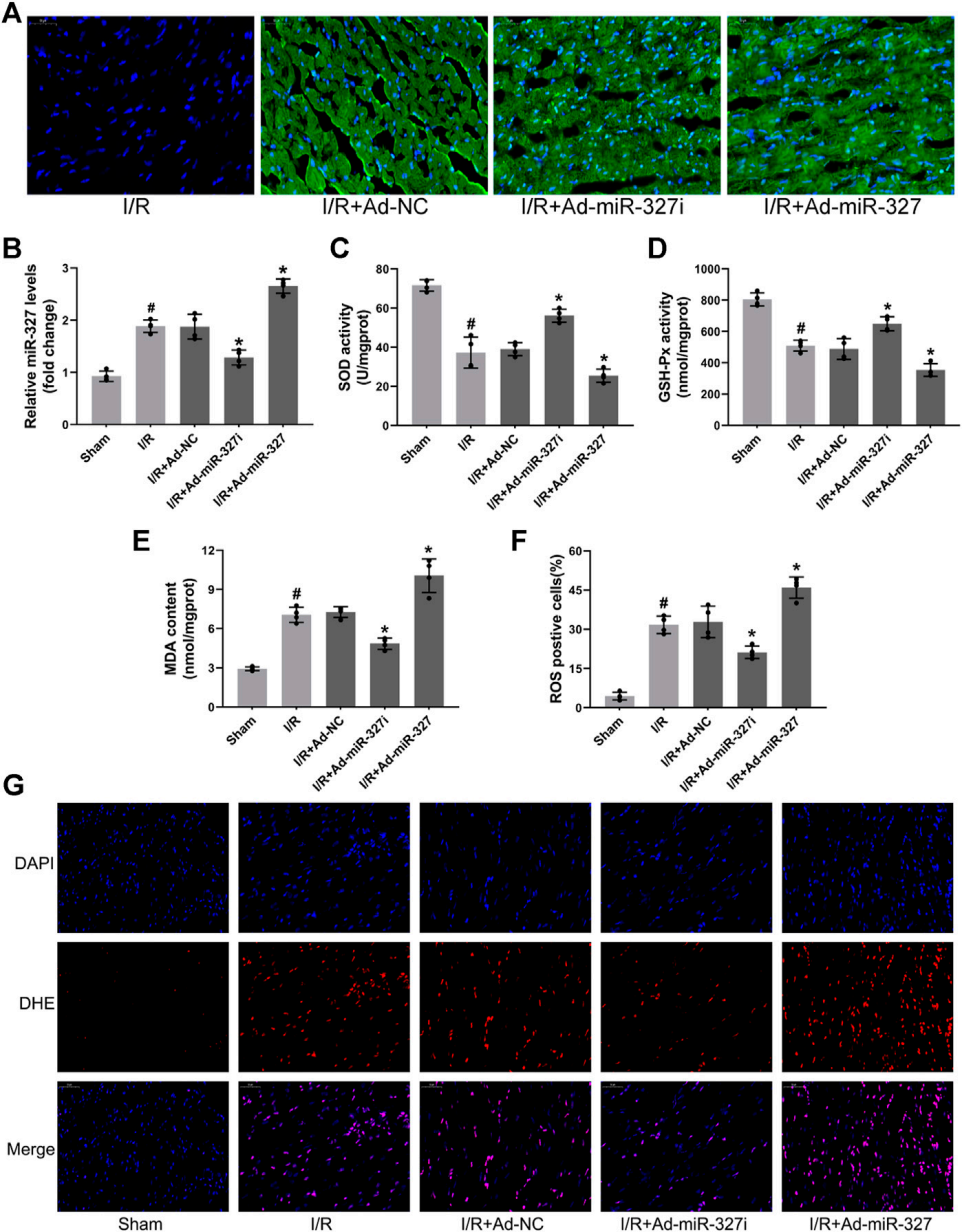
Effects of miR-327 on oxidative stress in MI/RI rats. Representative images of immunofluorescence microscopy after transfection of recombinant adenovirus or NS injection, DAPI-labeled nuclei of cardiomyocytes (blue), EGFP(green) and merged **(A)**. Relative expression of miR-327 in MI/RI rats **(B)**. The levels of SOD **(C)**, GSH-Px **(D)**, and MDA **(E)** in heart tissues of MI/RI rats. DHE staining for detecting intracellular ROS generation in heart tissues of MI/RI rats **(G)** in each group, and the analysis of ROS positive cells by Image-Pro Plus **(F)**. Data are expressed as the mean ± SD (*n* = 4); #*p* < 0.05 versus the Sham group; *p < 0.05 versus the Ad-NC group.

Initially, we examined the effect of miR-327 downregulation on oxidative stress induced by MI/RI, after 30 minutes ischemia followed by 2 h reperfusion (Li et al., 2018). Transfection of recombinant adenovirus vector induced obvious expression of green fluorescence protein in the myocardium and significantly regulated miR-327 using Ad-miR-327 or Ad-miR-327i, which provided basis for further experiments *in vivo* (Figures 8A,B). Ad-miR-327i obviously suppressed cardiac oxidative stress by heightened SOD and GSH-Px activities, and decreased MDA formation compared to Ad-NC (Figures 8C–E). In addition, the proportion of ROS-positive cells in the Ad-miR-327 group was obviously decreased (Figures 8F,G). However, Ad-miR-327 further aggravated oxidative damage in the heart after I/R compared to Ad-NC.

### Downregulation of miR-327 Alleviated MI/RI

**FIGURE 9:**
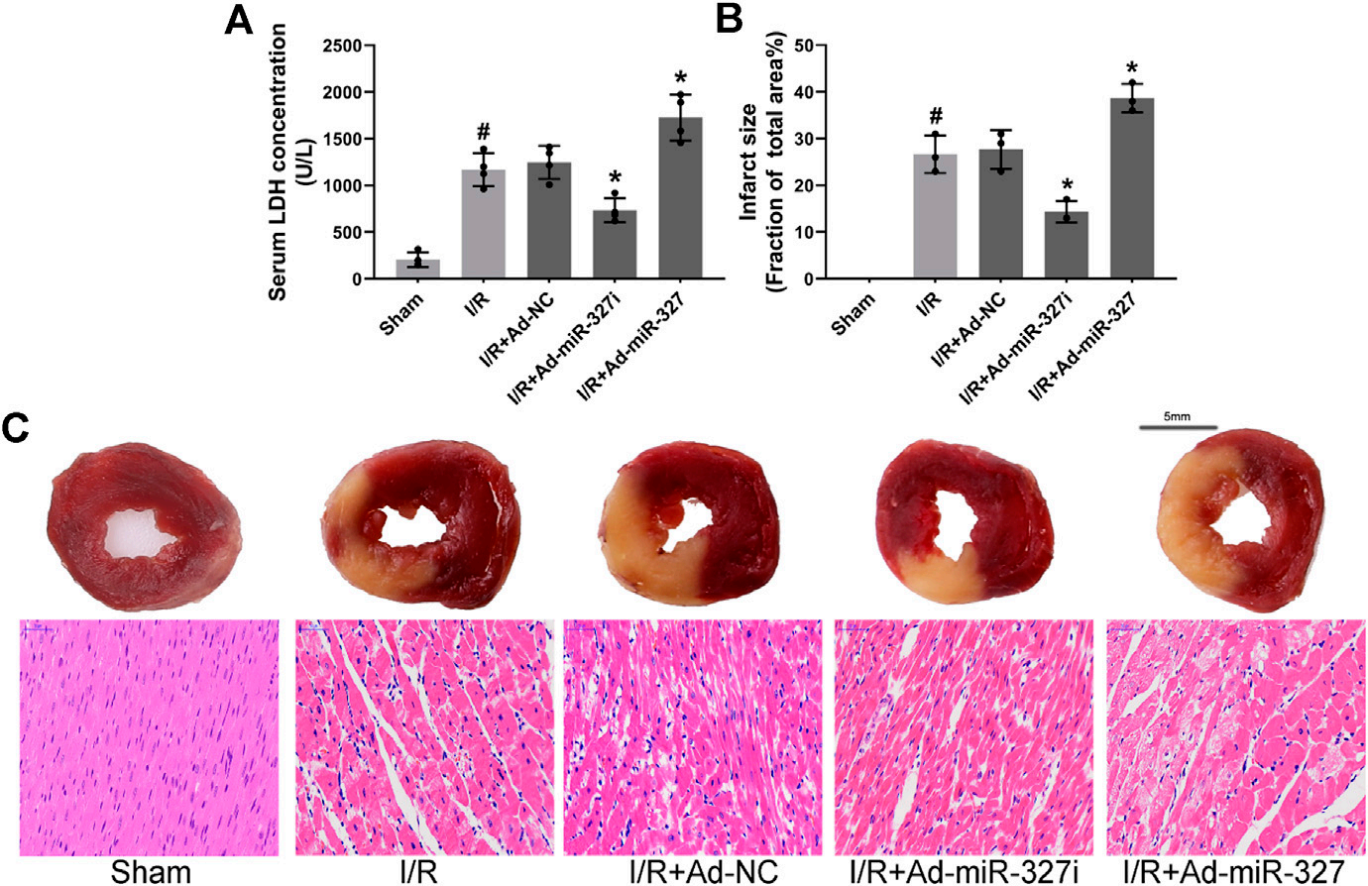
Effects of miR-327 on MI/RI in rats. Serum LDH concentration (*n* = 4) in each group **(A)**. Assessment of myocardial infarction by TTC staining (*n* = 3) and HE staining. For TTC staining, the red area represents non-infarcted tissue, while the white area represents infarcted tissue **(C)**, and myocardial infarct size expressed as (white area)/(white area + red area) (**B)**. Representative images of HE staining in each group **(C)**. Data are expressed as the mean ± SD; #*p* < 0.05 versus the Sham group; *p < 0.05 versus the Ad-NC group.

Then, we confirmed the effect of miR-327 downregulation on myocardial injury after I/R. After reperfusion, LDH concentration was obviously decreased in Ad-miR-327i rats compared with those in Ad-NC rats (734.75 ± 129.70 vs. 1246.50 ± 177.29 U/L), while it increased significantly in Ad-miR-327 rats (1727.50 ± 245.21 vs. 1246.50 ± 177.29 U/L; Figure 9A). In addition, Ad-miR-327 led to a dramatic increase in myocardial infarct size, while Ad-miR-327i relieved the damage to heart tissues, as demonstrated by a lower infarction volume than Ad-NC (Figures 9B,C). Collectively, these findings strongly supported that downregulation of miR-327 alleviated I/R-induced myocardial damage.

### MiR-327 Altered the FGF10/Akt/Nrf2 Signaling Pathway *In Vivo*

**FIGURE 10:**
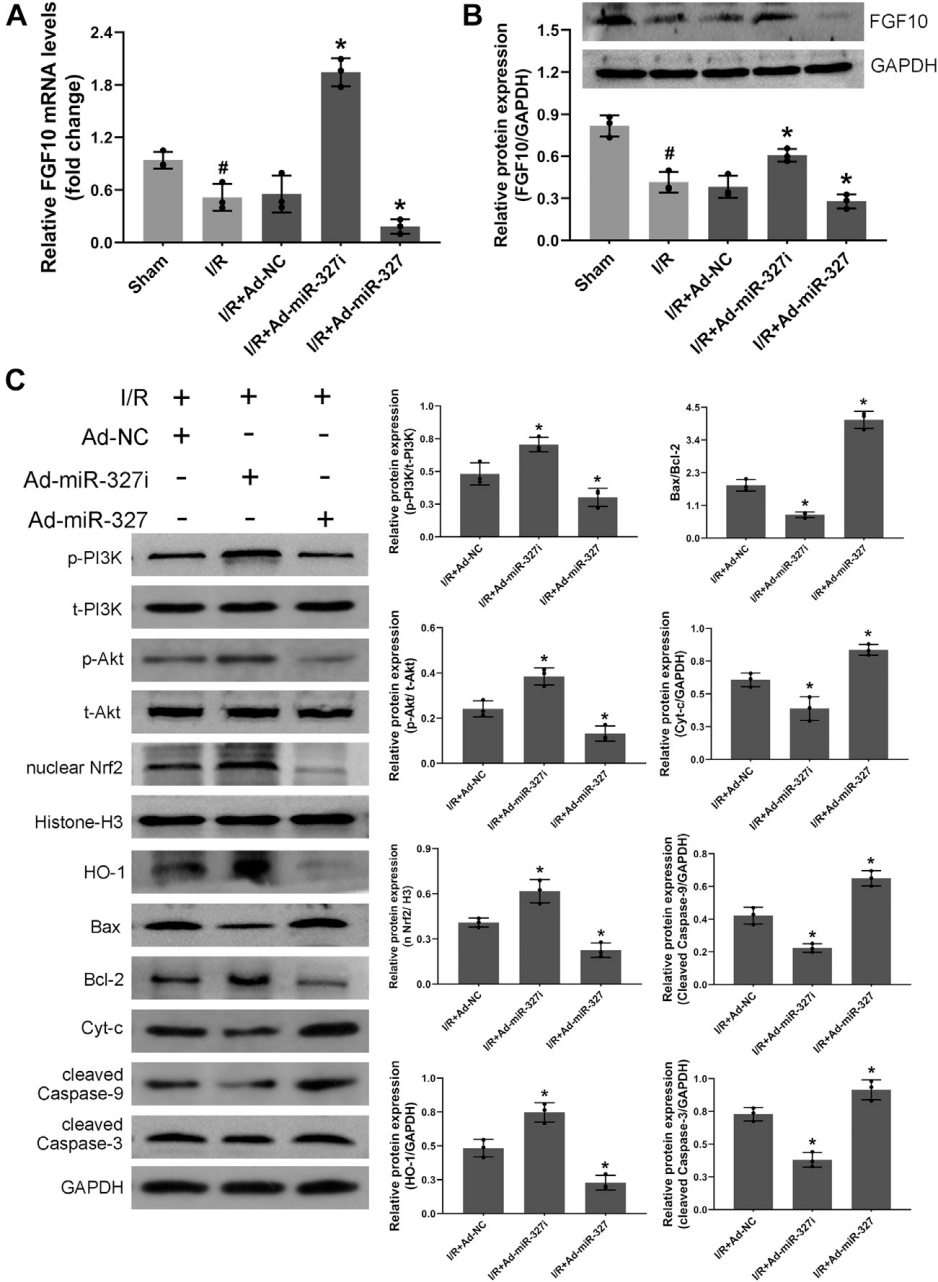
Effects of miR-327 on the FGF10/Akt/Nrf2 signaling pathway in MI/RI rats. FGF10 mRNA and protein levels were measured by qRT-PCR and the Western blot analysis in MI/RI rats heart tissues **(A-B)**. The proteins of p-PI3K and t- PI3K, t-Akt and *p*-Akt, nucleus Nrf2, HO-1, Bax, Bcl-2, Cyt-c, cleaved caspase-9, and cleaved caspase-3 in heart tissues were detected by the Western blot analysis **(C)**. Data are expressed as the mean ± SD (*n* = 3); #*p* < 0.05 versus the Sham group; *p < 0.05 versus the Ad-NC group.

Consistent with the results *in vitro*, downregulation of miR-327 obviously enhanced the mRNA and protein expression of FGF10 (Figures 10A,B). Moreover, the protein level of p-PI3K/t-PI3K, *p*-Akt/t-Akt, nucleus Nrf2, and HO-1 was elevated, while the expressions of Bax/Bcl-2, Cyt-c, cleaved caspase-9, and cleaved caspase-3 were reduced in the Ad-miR327i group. However miR-327 overexpression displayed opposite effects (Figure 10C). These findings suggested that downregulation of miR-327-suppressed oxidative stress may be related to activation of the FGF10/Akt/Nrf2 signaling pathway.

## Discussion

In the present study, we demonstrated that miR-327 participated in oxidative stress induced by myocardial I/R both *in vivo* and *in vitro*. Our data showed that myocardial I/R or TBHP stimulation promoted the excessive intracellular ROS generation that led to oxidative stress, deteriorated the cardiomyocyte, and induced the significant increase of miR-327 expression, which suggests that the increased expression of miR-327 in response to MI/RI may be partly due to ROS stimulation. Importantly, downregulation of miR-327 attenuated oxidative stress induced by myocardial I/R, whereas overexpression of miR-327 obviously aggravated oxidative damage. Hence, we suggest that miR-327 may be a mediator of oxidative stress during MI/RI, provided a therapeutic target for clinical treatment against MI/RI.

Some microRNA expression altered during myocardial I/R. In this study, we found that the expression of miR-327 in the myocardial tissue was significantly increased after I/R, which was also consistent with the results of systemic analyses of miRNA array in the I/R model of rat by Mukhopadhyay et al. (Mukhopadhyay et al., 2012). Moreover, the qPCR analyses demonstrated that the miR-327 expression was elevated about 3-fold in H9c2 cells under TBHP condition. Interestingly, it is reported that a number of specific miRNAs can be modulated by ROS (Carbonell and Gomes, 2020). A previous study showed that in H_2_O_2_-induced oxidative stress of rat neonatal cardiomyocytes, NF-κB, which as a ROS-sensitive transcription factor, could directly bind to the miR-21 promoter region to promote its expression (Wei et al., 2014). This also partly explained the reason for the elevated expression of miR-21 in response to H_2_O_2._ Unfortunately, the present study mainly focused on the role of miR-327 on oxidative stress induced by myocardial I/R, we will investigate whether the elevated expression of miR-327 is associated with these ROS-sensitive transcription factors in the next period, and look for potential reasons for the increased expression of miR-327 during MIRI.

MI/RI is a complex process involving inflammatory reactions, oxidative stress, and calcium overload, among which the subsequent reperfusion caused excessive ROS generation as a key factor contributed to reinjury of infarcted myocardium (Zhai et al., 2017; Li et al., 2018). Through transcriptomic analysis in the mouse MIRI model, Li et al. (Li et al., 2019) revealed that the changes in mitochondrial release of Cyt-c and oxidation–reduction process were the most obvious in myocardial tissue during the early reperfusion period (1–6 h). The ROS burst not only led to mitochondrial dysfunction and DNA damage but also triggered apoptosis, facilitated endoplasmic reticulum stress, and further aggravated the injury progression after myocardial I/R (Zhang et al., 2020). We evaluated oxidative stress by detecting intracellular ROS production, the activities of antioxidant enzymes SOD and GSH-Px, and MDA concentration (Zhao et al., 2018). Our study showed that myocardial I/R led to a great increase of ROS generation, inducing oxidative stress and resulting in cardiomyocyte damage, which was consistent with the previous research (Bian et al., 2018; Li et al., 2018). Furthermore, we confirmed that downregulation of miR-327 reduced ROS production, inhibited oxidative stress, and alleviated cardiomyocyte injury both in the H/R model of H9c2 cells and the MI/RI model of rats. However, overexpression of miR-327 showed severe oxidative damage. These results further verified that downregulation of miR-327 could alleviate myocardial ischemia/reperfusion injury and play a cardioprotection role by mediating oxidative stress.

In parallel, Ji et al.(Ji et al., 2018) reported that the miR-327 expression was the highest in fibrotic heart tissue induced by transverse aortic constriction (TAC) in mice, and downregulation of miR-327 inhibited cardiac hypertrophy and fibrosis by targeting integrin β3(ITGB3). In addition, some studies reported that excessive ROS can activate MMPs, which play an important role in LV remodeling (Hori and Nishida, 2009; Baghirova et al., 2016). It would be very meaningful to identify whether miR-327 is related to the postinfarction remodeling in the future.

TBHP, as a common alkyl hydrogen organic peroxide, induced massive hydrogen peroxide production including ROS in cells, which is often used to establish the model of oxidative stress damage in H9c2 cells (Fan et al., 2020). In the present study, the H9c2 cells employed are derived from the ventricular tissue of an embryonic BD1X rat, it does not have some characteristics of primary cardiomyocytes, such as spontaneous beats, but H9c2 cells also present morphological features of oxidative stress and apoptosis in response to H/R or TBHP stimulation (Kimes and Brandt, 1976; Kuznetsov et al., 2015). Bi et al. (Bi et al., 2018) showed that incubated H9c2 cells with 75 µM TBHP lead to a reduction of cell viability to about 54.61%, thereby establishing a model of oxidative stress–induced apoptosis. Besides, in another model of TBHP-induced oxidative stress, 50 µM TBHP stimulation for 3 h in H9c2 cells induced a remarkable elevate in intracellular ROS and triggered the expression of proapoptotic proteins (Silva et al., 2010). In this study, we found that the H9c2 viability decreased to about 58.84% at a 50 µM TBHP concentration after 4 h stimulation; meanwhile, the intracellular ROS level was also elevated more than 3-fold. The aim of present research was to explore the role of miR-327 on oxidative stress as well as apoptosis, so, this concentration of TBHP was considered to be appropriate in subsequent experiments.

Excessive ROS directly caused mitochondrial dysfunction, leading to Bax relocated on the mitochondria surface, promoting Cyt-c release, and activating caspase-9, and thus triggered the intrinsic apoptosis (Liang et al., 2017). Consistently, we observed that TBHP induced oxidative stress and promoted intrinsic apoptosis in H9c2 cells. It may also explain that the initiation of intrinsic apoptosis in the early phase of myocardial I/R may be partly due to the excessive ROS production. Results in TBHP-treated H9c2 cells have shown that downregulation of miR-327 repressed oxidative stress, decreased ROS-induced release of Cyt-c and the expression levels of Bax, and cleaved caspase-9. Besides, results *in vivo* have confirmed the effects of miR-327 on oxidative stress and intrinsic apoptosis, and further enhanced the understanding of the role of miR-327 in MI/RI.

FGF10, one of the FGF family members, is essential for developing embryo of cardiomyocytes (Rochais et al., 2014). Previous studies have shown that overexpression of FGF10 in adult mice cardiac tissue promotes cardiomyocyte cell-cycle reentry, suggesting that FGF10 might be a potential target for myocardial repair (Rochais et al., 2014). Wang et al. (Wang et al., 2021), performed a mouse myocardial infarction model, increased the FGF10 expression in the ischemic area of myocardium by directly injecting FGF10 coacervate, and found that myocardial injury was alleviated and cardiac function was improved. Differently, microRNAs, as endogenous small oligonucleotides, have shown a formidable role on gene expression regulation. Fischer et al. (Fischer et al., 2017) have confirmed that miR-327 targeted binding to FGF10 3′-UTR by the luciferase assay. Furthermore, we revealed that downregulation of miR-327 markedly promoted both mRNA and protein levels of FGF10, while miR-327 overexpression obviously suppressed FGF10 expression in H/R H9c2 cells and myocardial I/R rats. Therefore, we speculated that miR-327 directly regulated the FGF10 expression in MIRI. The combination of FGF10 and FGFR2b stimulates the intracellular FGF receptor substrate 2, which further activated the PI3K/Akt signaling pathway (Nakao et al., 2013). The *in vitro* and *in vivo* experiments showed that the elevated expression of FGF10 was accompanied by increased phosphorylation of PI3K and Akt, indicating that miR-327 inhibition attenuated MI/RI through, at least partially, the modulation of the FGF10-triggered PI3K/Akt signaling pathway.

In the current study, we have demonstrated that downregulation of miR-327 enhanced the activity of antioxidant enzymes, alleviated oxidative damage as well as apoptosis, but the mechanism that mediates this effect has not been revealed yet. Dong et al. (Hori and Nishida, 2009; Baghirova et al., 2016) documented that FGF10 plays an antioxidant role in peripheral nerve injury mainly dependent on enhanced expression of Nrf2/HO-1 activated by the PI3K/Akt signaling. Nrf2, as a key regulator of redox balance, enters into the nucleus to combine with ARE to trigger the transcription of endogenous protective genes (Bellezza et al., 2018). Evidence indicated that the PI3K/Akt pathway is involved in the activation of Nrf2, while inhibition of PI3K/Akt weakened the Nrf2 transcription activity (Chartoumpekis et al., 2010). In this work, our results showed that downregulation of miR-327 enhanced FGF10 expression and activated PI3K/Akt signaling, accompanied by an increase in nucleus Nrf2 and its downstream antioxidant factor HO-1 during myocardial I/R, H/R, and TBHP treatment. However, the level of Nrf2 transferring from the cytoplasm to nucleus was weakened, and HO-1 was decreased after upregulation of miR-327 in response to myocardial I/R and H/R. These findings suggested that downregulation of miR-327 activated Nrf2 through FGF10/Akt signaling transduction. In addition, the *in vitro* TBHP experiment showed that miR-327 knockdown inhibited intrinsic apoptosis, which might be attributed to the activation of the FGF10/Akt/Nrf2 signaling pathway. Collectively, the current study suggested that the miR-327–mediated FGF10/Akt/Nrf2 signaling pathway may play an important role in oxidative stress.

However, our present work has some limitations. First, in the present study, only one kind of cell line, the H9c2 cell, was used. Although H9c2 cell was widely used for the study of heart diseases, it cannot fully represent the primary cardiomyocytes. Second, TBHP promoted the miR-327expression, but the mechanism leading to this effect has not been clear. Third, the effect of different TBHP concentrations on cell death was not the same, so the effect of miR-327 on cell survival under high-concentration TBHP stimulation needs to be further explored. Fourth, miR-327 has been reported to target different genes to exert biological functions, but the role of microRNA on gene expression is not only in the form of negative regulation at the posttranscriptional level (Dragomir et al., 2018); other regulatory mechanisms on miR-327 also merit future investigation.

In conclusion, the inhibition of miR-327 could alleviate oxidative stress induced by MI/RI. And the mechanism that mediates this effect is possibly by regulating the FGF10/Akt/Nrf2 pathway, which could be a promising therapeutic agent for MIRI.

## Data Availability

The raw data supporting the conclusion of this article will be made available by the authors, without undue reservation.
